# Conditioned place preference training prevents hippocampal depotentiation in an orexin-dependent manner

**DOI:** 10.1186/s12929-017-0378-0

**Published:** 2017-09-06

**Authors:** Guan-Ling Lu, Hau-Jie Yau, Lih-Chu Chiou

**Affiliations:** 1 Graduate Institute of Pharmacology, College of Medicine, National Taiwan University, Taipei, Taiwan; 2 Graduate Institute of Brain and Mind Sciences, College of Medicine, National Taiwan University, Taipei, Taiwan; 3 Reserach Center for Chinese Medicine & Acupuncture, China Medical University, Taichung, Taiwan; 4 Department of Pharmacology, College of Medicine, National Taiwan University, No. 1, Jen-Ai Rd., Section 1, Taipei, 100 Taiwan

**Keywords:** Orexin, Depotentiation, Conditioned place preference, Hippocampus

## Abstract

**Background:**

Long-term potentiation (LTP) is well recognized as a cellular-correlated synaptic plasticity of learning and memory. However, its reversal forms of synaptic plasticity, depotentiation, is less studied and its association with behaviors is also far from clear. Previously, we have shown that nanomolar orexin A can prevent the depotentiation induced by low frequency stimulation (LFS) following theta burst stimulation-induced LTP, namely inducing re-potentiation, at hippocampal CA1 synapses in vitro. Here, we explored the functional correlate of this orexin-mediated hippocampal re-potentiation.

**Methods and results:**

We found that intraperitoneal (*i.p.*) injection process-paired contextual exposures during the conditioned place preference (CPP) task in mice resulted in re-potentiation at CA1 synapses of hippocampal slices, regardless of whether the CPP behavior is expressed or not. Simply exposing the mouse in the CPP apparatus, or giving the mouse consecutive *i.p.* injections of saline in its home cage or a novel cage did not lead to hippocampal re-potentiation. Besides, this CPP training process-induced hippocampal re-potentiation was prevented when mice were pretreated with TCS1102, a dual orexin receptor antagonist. These results suggest that the expression of hippocampal re-potentiation is orexin-dependent and requires the association of differential spatial contexts and *i.p.* injections in the CPP apparatus.

**Conclusions:**

Together, we reveal an unprecedentedly orexin-mediated modulation on hippocampal depotentiation by the training process in the CPP paradigm.

**Electronic supplementary material:**

The online version of this article (10.1186/s12929-017-0378-0) contains supplementary material, which is available to authorized users.

## Background

Orexin A and orexin B [1], also named hypocretin 1 and hypocretin 2 [2], are a pair of neuropeptides derived from prepro-hypocretin, which is expressed only in the perifornical area and lateral hypothalamus (LH). There are two orexin receptors, OX_1_Rs and OX_2_Rs [3]. OX_1_Rs display similar affinity for orexin A and orexin B while OX_2_Rs have higher affinity for orexin B [1]. Both OX_1_Rs and OX_2_Rs are widely distributed throughout the brain [4], including the hippocampus [5], a crucial brain area involved in learning and memory [5–7] and exhibits several forms of synaptic plasticity, including long-term potentiation (LTP), long-term depression (LTD) and depotentiation [8]. LTP and LTD can be induced by high frequency stimulation (HFS) and low frequency stimulation (LFS), respectively. Both forms of hippocampal synaptic plasticity are believed to be the cellular correlates of memory and oblivion (forgetting), respectively [8]. Depotentiation is one form of LTD induced by LFS after HFS while its functional role remains unclear. Orexins have been shown to modulate LTP in the hippocampus in vivo and in vitro [5, 9–12]. Previously, we found that orexin A can attenuate theta bust stimulation (TBS)-induced LTP at micromolar concentrations while prevented LFS-induced depotentiation, namely inducing re-potentiation, at sub-nanomolar concentrations at Schaffer collateral-CA1 synapses of hippocampal slices, via OX_1_Rs and OX_2_Rs [12]. The latter re-potentiation effect of sub-nanomolar orexin can occur under physiological conditions. We, therefore, further explore its physiological significance, i.e. under what situations; orexins will be released to induce hippocampal re-potentiation.

Several lines of evidence indicate that orexins are involved in the conditioned place preference (CPP) induced by abusing substances [13, 14] or food [15]. CPP is a contextual learning paradigm requiring associative learning between the reward and its spatial context [16], and is wildly used to explore the reinforcing potential of abusing substances [17]. It is a hippocampus-dependent task since lesion and inactivation of dorsal hippocampus impaired food and cocaine CPP, respectively [18, 19]. Recently, the finding of Rashidy-Pour et al. [20] is especially noted that intra-hippocampal blocking OX_1_Rs or OX_2_Rs prevented LH stimulation-induced CPP. This suggests that endogenous orexins in the hippocampus are required for the development of CPP. Combining our previous finding that orexin induced hippocampal re-potentiation [12], we therefore hypothesized that CPP development can modify hippocampal synaptic properties in an orexin-dependent manner. Here, we have tested this hypothesis by examining the modulation of cocaine CPP on orexin-induced re-potentiation at Schaffer collateral-CA1 synapses of hippocampal slices. To our surprise, we found that orexin-induced hippocampal re-potentiation appears to be a shared cellular correlate specific to an association of intraperitoneal (*i.p.*) injections-paired contextual exposures during the CPP training process, regardless whether cocaine CPP is expressed or not. Our findings reveal an unprecedentedly cocaine-dissociated but CPP training process-specific and orexin-dependent modulation on hippocampal depotentiation.

## Methods

### Animals

All animal experiments adhere to the guidelines and were approved by the Institutional Animal Care and Use Committee of College of Medicine, National Taiwan University. Male C57BL/6JNarl mice were housed under a 12-h light/dark cycle in a climate-controlled room with ad libitum access for food and water. On the day for conducting in vivo experiments, mice were moved to and acclimated in the behavior room for at least 1 h. All efforts were made to minimize the number of animals used and their suffering.

### The CPP paradigm

**Fig. 1:**
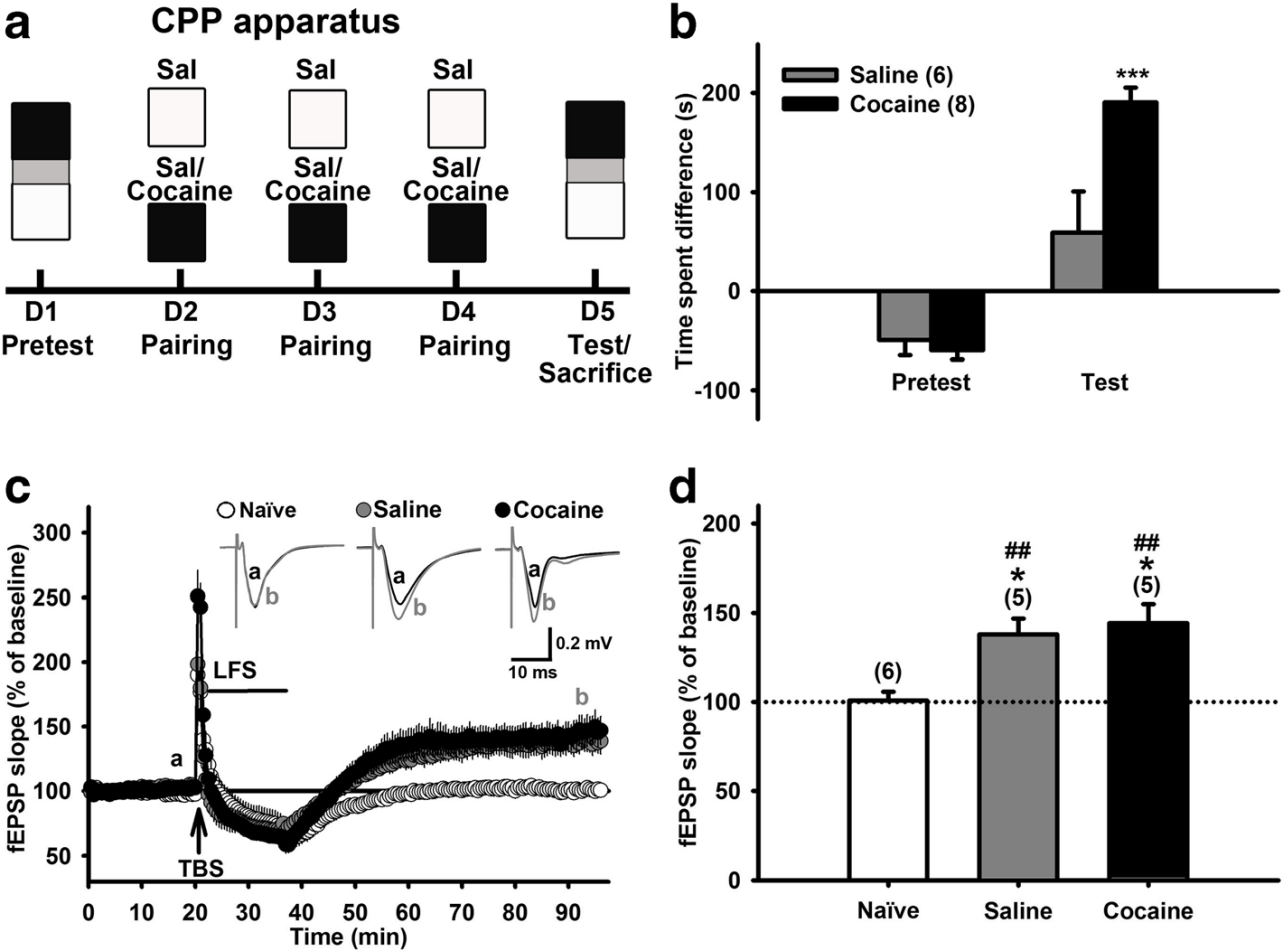
CPP training prevents LFS-induced hippocampal depotentiation in vitro. **a**: The schema for a 5-day bias cocaine CPP task with a 3-day training period. Cocaine CPP was induced by giving the mouse cocaine (20 mg/kg, *i.p.*) and saline (Sal) injections, respectively, in its non-preferred and preferred arenas in the CPP apparatus daily for three days. In the saline-conditioned group, cocaine injections were replaced by saline injections. **b**: Bar graphs summarize the CPP scores for saline- and cocaine-conditioned groups of mice. Note that cocaine-, but not saline-, conditioned mice developed significant CPP (***p < 0.001, saline-conditioned group vs. cocaine-conditioned group in the test stage, two-way ANOVA with Bonferroni post hoc test). **c**: Time courses of the slope of fEPSPs recorded before, during theta burst stimulation (TBS, arrow) and low frequency stimulation (LFS, horizontal line), and after LFS in hippocampal slices prepared from naïve, saline- and cocaine-conditioned groups of mice after performing the CPP test on Day 5 . The slope of each fEPSP is expressed as % of the baseline fEPSP slope, which is the average of 20 fEPSPs recorded 10 min before TBS. Inset: Averaged traces of 20 fEPSPs recorded before (a) and 60 min after (b) LFS in each group. **d**: Bar graphs summarize the magnitudes of potentiation of fEPSP slope in naïve, saline-conditioned, and cocaine-conditioned groups. The magnitude of potentiation was calculated from the average of the slope of 20 fEPSPs recorded 50 ~ 60 min after LFS and expressed as % of baseline. The number in parentheses is the number of slices recorded. Data are mean ± S.E.M. *p < 0.05 vs. 100%, paired *t*-test. ^##^ *p* < 0.01 vs. the naïve group (one-way ANOVA with Newman-Keuls post-hoc analysis)

A bias CPP paradigm with a 3 day-training protocol was used to induce cocaine CPP in mice (8–12 weeks) as described previously [14] and as shown in Fig. 1a. Briefly, on Day 1 (the pretest stage), the mouse was allowed to move freely for 10 min in the CPP apparatus, which consists of black and white arenas with different floor features separated by an intermediate compartment. The time that each mouse spent in each arena was used for grouping the mice with approximately equal bias of black or white arena preference. Mice were excluded if they spent more than 4 min in the neutral chamber or the time spent difference between white and black chambers was greater than 100 s. On each day of Days 2–4, the mouse was given an *i.p.* injection of saline and placed in its preferred arena for 30 min. Six hours later, the same mouse was given cocaine hydrochloride (20 mg/kg, *i.p.*) (the cocaine-conditioned group) or saline (the saline-conditioned group) and placed in its non-preferred arena for 30 min. Drug-paired arenas were randomized among all groups. On Day 5 (the test stage), the mouse was allowed to move freely in the CPP apparatus as the procedure on Day 1. The preference score in each mouse was calculated by subtracting the time spent in the saline-paired arena from the time spent in the cocaine-paired arena.

### Saline-conditioning in different contexts

To differentiate whether the change in hippocampal synaptic plasticity is CPP context-specific, we repeated the saline-conditioning procedure as in the CPP training process but changed the conditioning context. The CPP apparatus was replaced with the home cage (47*26*21 cm) of the tested mouse or a large novel bedded cage (30*19*13 cm).

### Electrophysiological recordings

Extracellular recordings of field excitatory postsynaptic potentials (fEPSPs) were performed at Schaffer collateral-CA1 synapses of mouse hippocampal slices (300 μm) with an MED64 multichannel recording system (Alpha MED sciences Co., Ltd., Tokyo, Japan) as described previously [12]. Briefly, the mouse was sacrificed after the behavioral operant and coronal hippocampal slices were dissected from the mouse and equilibrated at room temperature for at least 1.5 h in the artificial cerebral spinal fluid (aCSF). It consisted of (mM) NaCl 117, KCl 4.5, CaCl_2_ 2.5, MgCl_2_ 1.2, NaH_2_PO_4_ 1.2, NaHCO_3_ 25 and glucose 11. When performing recording, the slice was placed on a 8 × 8 multi-electrode dish probe (MED-P515A; Alpha MED sciences Co., Ltd.) and perfused with aCSF at the rate of 1.0 ~ 1.5 ml/min. One of 64 electrodes over the Schaffer collateral/commissural fiber path in the CA1 region was chosen as the stimulating point. The sharpest and largest fEPSP detected from one of other 63 electrodes in the CA1 stratum radiatum of each hippocampal slice was recorded. The slope of the fEPSP was measured via the Conductor® software.

fEPSPs were evoked at 0.03 Hz and the slope of every fEPSP, which represents the synchronization of postsynaptic responses upon presynaptic stimulation and reflects the magnitude of synaptic transmission, was recorded and the average of 20 fEPSP slopes recorded 10 min before LTP was taken as the baseline of synaptic transmission. LTP and depotentiation of fEPSPs were induced and analyzed as reported previously [12]. LTP was induced by TBS [12, 21], and its magnitudes was measured by the averaged slope of 20 fEPSPs recorded 50 ~ 60 min after TBS. Depotentiation was induced by LFS (1 Hz, 15 min) at 1 min after TBS, and its magnitude was measured by the averaged slope of 20 fEPSPs recorded 50 ~ 60 min after LFS, and expressed as % of the baseline slope of fEPSPs.

### Drugs

Cocaine hydrochloride was purchased from the Division of Controlled Drugs, Food and Drug Administration, Department of Health, Executive Yuan, Taiwan. TCS1102, *N*-[1,1′-Biphenyl]-2-yl-1-[2-[(1-methyl-1*H*-benzimidazol-2-yl)thio]acetyl-2-pyrrolidinedicarboxamide, was purchased from Tocris Bioscience (Bristol, UK). For in vivo studies, cocaine hydrochloride was dissolved in 0.9% sodium chloride (NaCl). TCS1102 was dissolved in 0.9% normal saline containing 50% *v*/v PEG-200.

### Statistical analysis

Data are presented as the mean ± S.E.M. The n and N numbers indicate the number of tested slices (n) and animals (N), respectively. Two-way repeated-measures Analysis of Variance (ANOVA) with Bonferroni’s post hoc test was used for the comparison in the CPP test. For electrophysiological data, statistical comparison was performed by one-way ANOVA with the Newman-Keuls multiple comparison test for groups of 3 or more, and Student’s *t*-test or paired *t*-test was used for groups of 2. *P* < 0.05 was considered to be of significant difference.

## Results

### LFS failed to induce hippocampal depotentiation in both cocaine- and saline-conditioned groups in the CPP paradigm

Fig. 1a depicts the protocol of a 3-day cocaine CPP training paradigm in mice. The result showed that cocaine-conditioned, but not saline-conditioned, mice had a significantly higher CPP score on Day 5 (the test stage) than that on Day 1 (the pretest stage) (Fig. 1b), suggesting this task paradigm has successfully established cocaine CPP in mice. A two-way ANOVA with repeated measures showed a significant interaction between treatment and stage [F_(1,12)_ = 14.4, *p* = 0.003]. Post hoc Bonferroni analysis showed a significance difference in CPP scores between saline- or cocaine-conditioned groups (*p* < 0.001).

After the CPP test, hippocampal slices were prepared from these two groups of mice for extracellular recordings of synaptic plasticity. In slices isolated from naïve mice, the average slope of the fEPSP, as measured in the last 10 min of the 60 min recording period following LFS, was not significantly different from that in the baseline period [Fig. 1c and d, 100.5 ± 4.1%, N (number of animals) = 6, n (number of slices) = 6, *p* = 0.9, paired *t*-test]. This suggests LFS completely attenuates TBS-elicited LTP, in the hippocampal Schaffer collateral-CA1 pathway of naïve mice, as reported in our previous study [12], i.e. LFS successfully induce depotentiation in the naïve group. On the other hand, in slices from cocaine-CPP mice, LFS failed to induce depotentiation (Fig. 1c and d). To our surprise, LFS also failed to induce depotentiation in slices from the saline-conditioned group (Fig. 1c and d). This suggests that despite the mice learned to distinguish cocaine from saline administration after conditioning (Fig. 1b), LFS failed to induce depotentiation in these mice, regardless of saline or cocaine were conditioned (Fig. 1c and d). The average fEPSP slopes of saline- and cocaine-conditioned groups were 137.9 ± 8.9% (N = 5, n = 5, *p* = 0.013, paired *t*-test) and 144.2 ± 10.6% (N = 5, n = 5, *p* = 0.014, paired *t*-test) of baseline, respectively (Fig. 1c). One-way ANOVA showed a significant difference among these three groups [F_(2,13)_ = 9.3, *p* = 0.003]. A post hoc Newman-Keuls multiple comparison showed a significance difference between naïve and saline-conditioned groups as well as naïve and cocaine-conditioned groups (*p* < 0.01 and p < 0.01, respectively) (Fig. 1d).

The failure of LFS to de-potentiate TBS-induced LTP suggests the existence of an antagonizing mechanism of depotentiation, named “re-potentiation” [12]. Naïve mice did not receive the CPP training and displayed de-potentiation (i.e. no re-potentiation occurred). However, the saline group did receive the CPP training but did not develop CPP, and did not display de-potentiation (i.e. generating re-potentiation). The cocaine group developed CPP and generated re-potentiation. Therefore, the re-potentiation (as seen in the saline- and cocaine-paring groups) is dependent on the CPP training process, but independent of the establishment of CPP. Therefore, this “re-potentiation” following TBS-LFS at the hippocampal Schaffer collateral-CA1 pathway is dissociated from pharmacological effects of cocaine and independent on the establishment of CPP.

### Saline or cocaine injections in home-caged mice did not induce re-potentiation

**Fig. 2:**
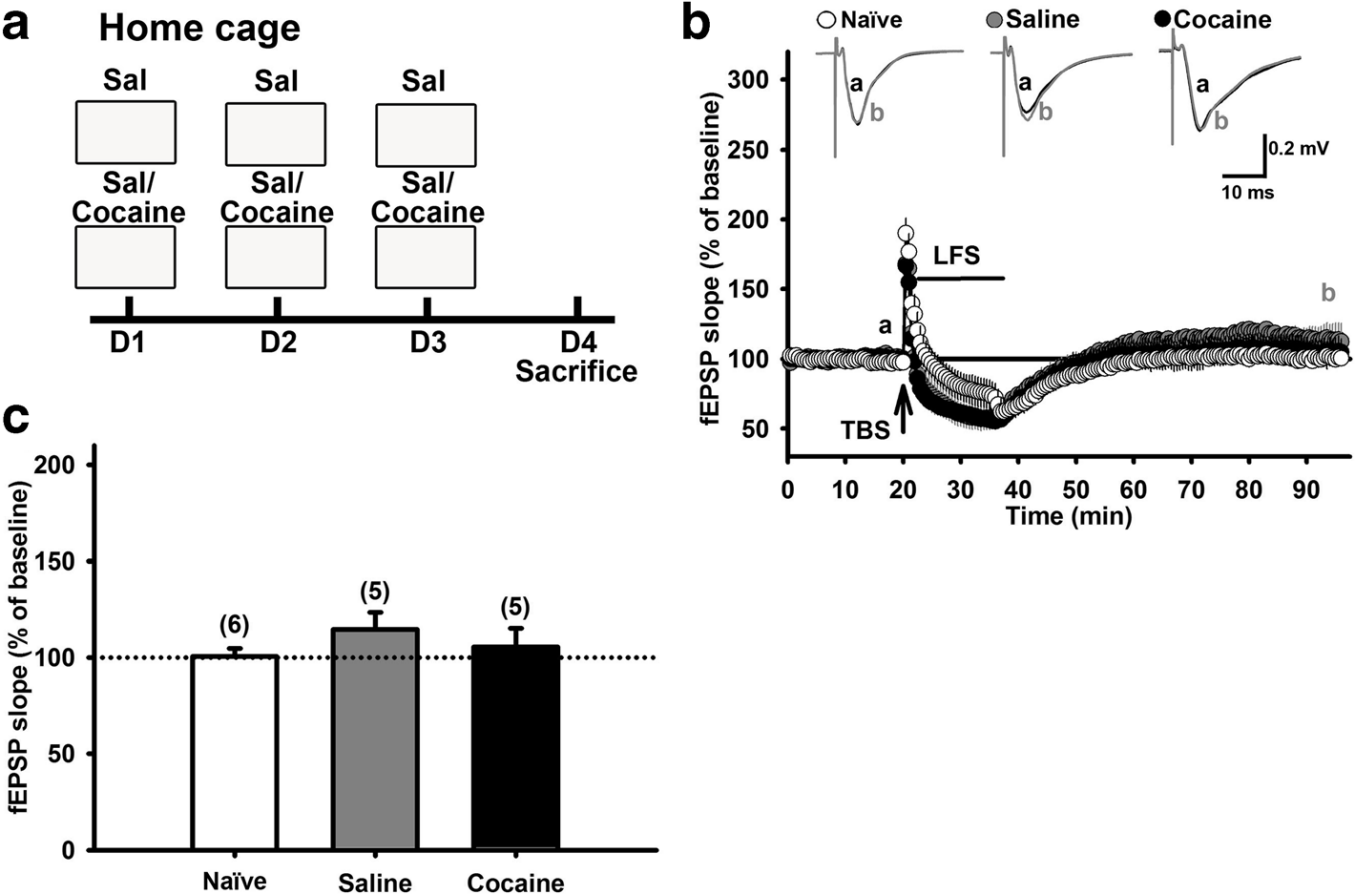
Home cage pairings with saline or cocaine injections in mice failed to prevent LFS-induced hippocampal depotentiation in vitro. **a**: The schema for a 3-day home cage parings with saline and cocaine (20 mg/kg, *i.p.*) injections under the same protocol as used in the CPP training stage. Hippocampal slices were prepared on Day 4. **b**: Time courses of the slope of fEPSPs recorded before, during TBS and LFS, and after LFS in hippocampal slices prepared from untreated naïve mice and mice receiving saline or cocaine injections at their home cage. Inset: Averaged traces of 20 fEPSPs recorded before (a) and 60 min after (b) LFS in each group. **c**: Bar graphs summarize the magnitudes of potentiation of fEPSP slope in the three groups of mice in **b**. Depotentiation was induced, analyzed and presented as described in Fig. 1

To further examine whether the observed hippocampal re-potentiation is a context-specific phenomena, we performed the CPP training process in the mouse home cage. Two groups of mice received the same 3-day consecutive *i.p.* saline and cocaine injections, respectively, at their home cages as in the CPP training process. Mice were sacrificed on Day 4 and their hippocampal slices were isolated (Fig. 2a). In home-caged mice, LFS was not able to induce hippocampal re-potentiation in both either saline- or cocaine-injection groups (Fig. 2b, c). The average fEPSP slopes of saline- and cocaine-injection groups were 114.5 ± 9.0% (N = 5, n = 5, *p* = 0.18, paired *t*-test) and 105.4 ± 9.7% (N = 5, n = 5, *p* = 0.61, paired *t*-test) of baseline, respectively (Fig. 2c). One-way ANOVA showed no significant difference among naïve, saline-injection and cocaine-injection groups [F_(2,13)_ = 0.87, *p* = 0.44] (Fig. 2c). The home-caged mice in either saline- or cocaine-injection group were able to show LTP following TBS, as in the naïve group (the saline group: 147.3 ± 6.7% of baseline, N = 4, n = 6; the cocaine group: 161.3 ± 8.4% of baseline, N = 3, n = 6; the naïve group: 141.9 ± 4.3% of baseline, N = 6, n = 6; Additional file 1: Figure S1). Thus, the failure to induce re-potentiation is not due to that the hippocampal Schaffer collateral-CA1 synapses of these mice were unable to potentiate. These results suggest that the injection process-paired contextual exposure in the CPP apparatus is important for developing re-potentiation at hippocampal Schaffer collateral-CA1 synapses.

### The injection procedure-paired contextual exposures in the CPP apparatus is required for re-potentiation

**Fig. 3:**
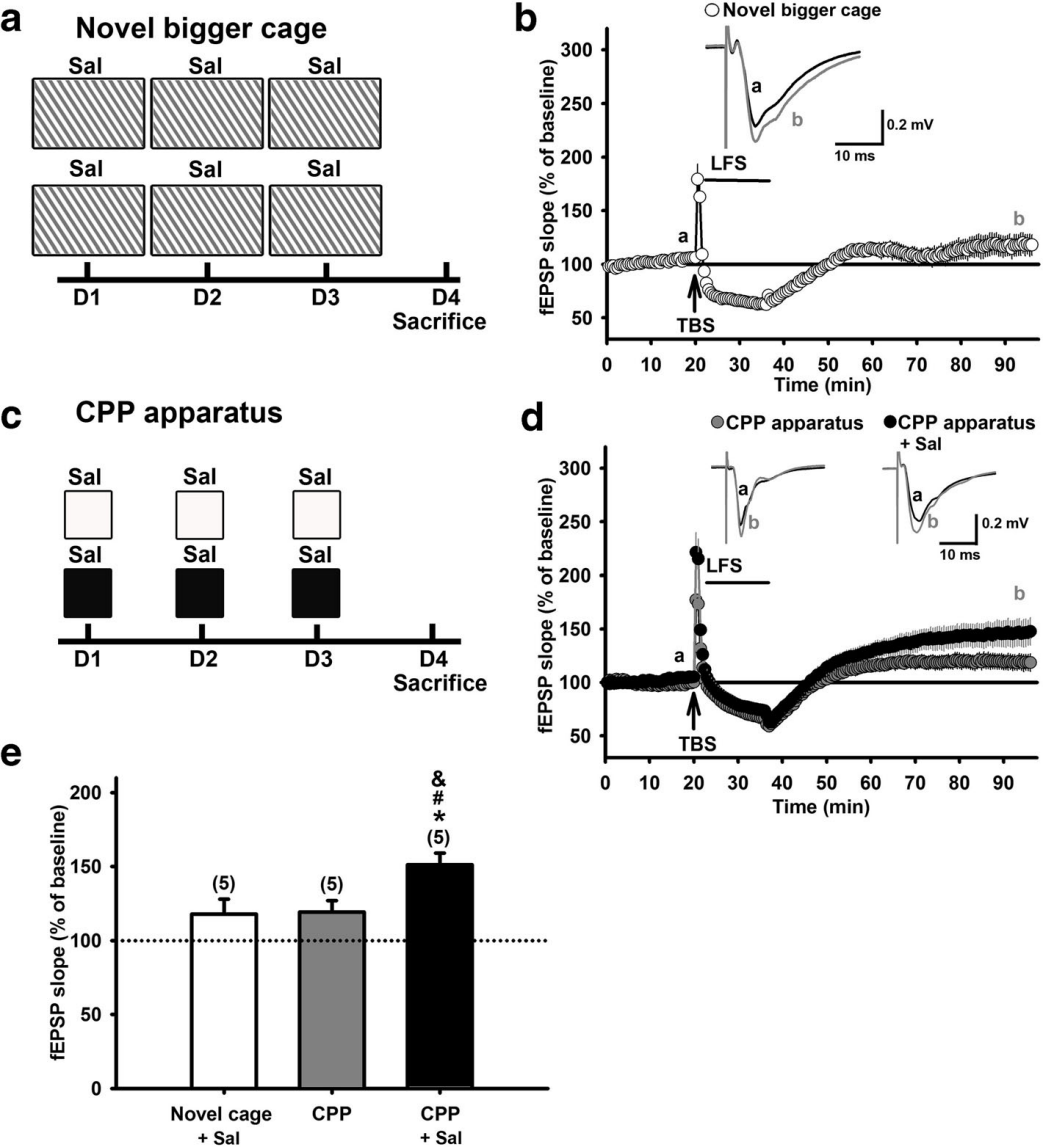
Novel cage pairing with saline injections or injection-free CPP training failed to prevent LFS-induced hippocampal depotentiation in vitro. **a**: The schema for a group of mice given saline injections twice a day in a novel big cage for 3 days. Hippocampal slices were prepared on Day 4. **b**: Time courses of the slope of fEPSPs recorded before, during TBS and LFS, and after LFS in hippocampal slices prepared from mice underwent procedures in A. Inset: Averaged traces of 20 fEPSPs recorded before (a) and 60 min after (b) LFS. **c**: The schema for mice underwent saline-paired CPP training or *i.p.* injection-free CPP training. Hippocampal slices were prepared on Day 4. **d**: Time courses of the slope of fEPSPs recorded before, during TBS and LFS, and after LFS in hippocampal slices prepared from the two groups of mice in **c**. Inset: Averaged traces of 20 fEPSPs recorded before (a) and 60 min after (b) LFS in each group. **e**: Bar graphs summarizes the magnitudes of potentiation of fEPSP slope in the three groups of mice in **a** (Novel cage + saline) and **c** (*i.p.*-free CPP and CPP + saline). Depotentiation was induced, analyzed, presented as described in Fig. 1. *p < 0.05 vs. 100%, paired *t*-test. ^#^ *p* < 0.05 vs. the novel cage + saline group and ^&^ *p* < 0.05 vs. the CPP group (one-way ANOVA with Newman-Keuls post-hoc analysis)

To examine if the novelty introduced by the contextual exposures in the CPP apparatus may contribute to the development of hippocampal re-potentiation, a larger novel cage was used to replace the familiar home cage for the conditioning process in the previous experiment. We found that the novelty introduced by a novel larger cage was not able to produce hippocampal re-potentiation (Fig. 3b and e, the average fEPSP slope was 118.0 ± 9.7% of baseline, N = 4, n = 5, *p* = 0.13, paired *t*-test) or to affect TBS-LTP (Additional file 1: Figure S2B, S2E). The failure to induce re-potentiation in the novel cage group is not due to the failure of LTP induction since the magnitude of LTP in this group is similar to that in the naïve group (the novel cage group: 153.1 ± 6.4% of baseline, N = 3, n = 6, *p* = 0.2, *t*-test *v.s.* naïve group).

We further investigated whether the association between *i.p.* injections and differential contextual exposures during the CPP training process is required and sufficient to induce hippocampal re-potentiation. In the experimental group, mice were daily exposed to white and black arenas of the CPP apparatus, respectively, and received saline injections in each arena for three days (Fig. 3c), as the 3-day training stage in the CPP task (Fig. 1a). On the other hand, the control group was exposed to the CPP apparatus only and did not receive saline injection. Interestingly, simply exposing the mice to the CPP apparatus not only did not develop a preference between black and white arenas (Additional file 1: Figure S3) and was not enough to produce hippocampal re-potentiation (Fig. 3d and e, the average fEPSP slope was 119.2 ± 7.5% of baseline, N = 4, n = 5, *p* = 0.06, paired *t*-test,). The failure to induce re-potentiation in this group is not due to the failure of LTP induction since it displayed significant LTP following TBS (Additional file 1: Figure S2D, S2E). Therefore, the association between contextual exposures and *i.p.* injections during CPP training is required to produce hippocampal re-potentiation (Fig. 3d and e, the average fEPSP slope was 151.1 ± 7.9% of baseline, N = 4, n = 5, *p* = 0.003, paired *t*-test).

Taken together, here we revealed that it is the association of repetitive differential context exposures and *i.p.* injections during the CPP training process that can enable hippocampal Schaffer collateral-CA1 synapses to resist LFS-induced depotentiation.

### Endogenous orexins are involved in CPP training-induced hippocampal re-potentiation

**Fig. 4:**
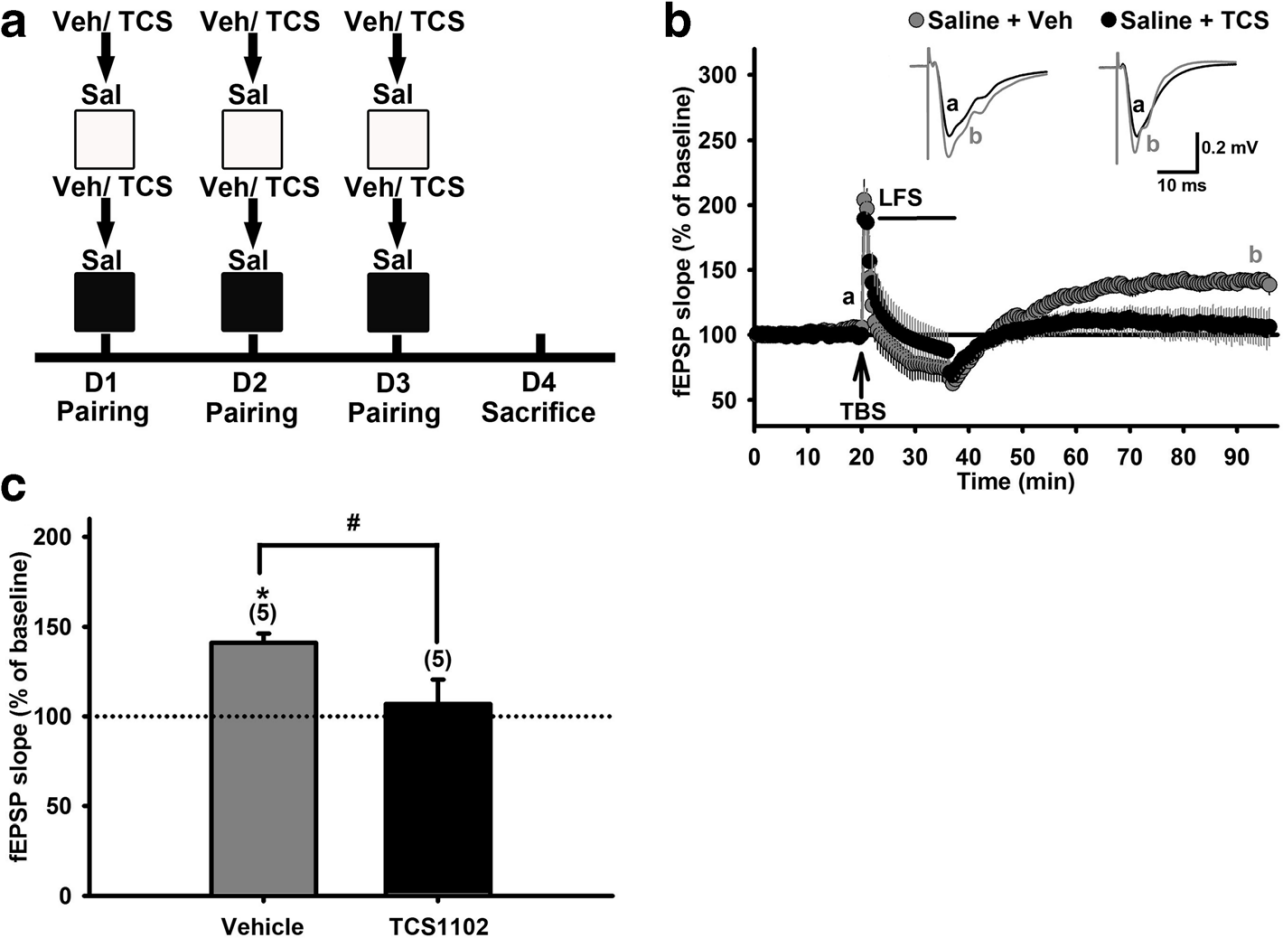
Pretreatment with TCS1102, a dual orexin receptor antagonist, restored LFS-induced hippocampal de-potentiation in vitro in mice underwent saline-paired CPP training. **a**: The schema for mice given vehicle (Veh) or TCS1102 (TCS, 20 mg/kg, *i.p.*) 30 min prior daily saline-paired CPP training process. Hippocampal slices were prepared on Day 4. **b**: Time courses of the slope of fEPSPs recorded before, during TBS and LFS, and after LFS in hippocampal slices prepared from the two groups in **a**. Inset: Averaged traces of 20 fEPSPs recorded before (a) and 60 min after (b) LFS in each group. **c**: Bar charts summarize the magnitudes of potentiation of fEPSP slope in vehicle- or TCS1102-treated mice. Depotentiation was induced, analyzed, presented as described in Fig. 1. *p < 0.05 vs. 100%, paired *t*-test. ^#^ *p* < 0.05 vs. the TCS group, un-paired *t*-test

Previously, we have demonstrated that sub-nanomolar orexin A can prevent LFS- induced hippocampal depotentiation in vitro and this effect is mediated by OX_1_Rs and OX_2_Rs [12]. We, therefore, are intrigued to examine whether endogenous orexins may mediate the CPP training-induced hippocampal re-potentiation described above. To validate this hypothesis, mice were pretreated with a dual OXR antagonist, TCS1102 (20 mg/kg, *i.p.*), 30 min before undergoing the CPP training process (Fig. 4a). TCS1102 and its vehicle pretreatments did not affect the time spent difference between white and black arenas in mice (Additional file 1: Figure S4). However, TCS1102 pretreatment significantly inhibited CPP training-induced hippocampal re-potentiation (Fig. 4b and c; the average fEPSP slope of the baseline: TCS1102 group, 141.0 ± 5.3%, N = 4, n = 5; vehicle group, 106.8 ± 13.7%, N = 3, n = 5; *p* = 0.047, unpaired *t*-test).

These results suggest that endogenous orexins are released during the CPP training process, even without developing CPP, can prevent LFS from inducing de-potentiation at Schaffer collateral-CA1 synapses via OXRs; i.e. keeping synaptic transmission remained potentiated.

## Discussion

The present study investigated the modulation of CPP task on the hippocampal synaptic properties, specifically assessed by an orexin-dependent hippocampal re-potentiation. During CPP training, the mouse was exposed to two different arenas of the CPP apparatus and passively given an *i.p.* injection in each arena for three consecutive days. Although the mice showed behavioral preference to the arena previously paired with cocaine *i.p.* injection, we did not detect a cocaine-specific synaptic adaption in hippocampal CA1 synapses, assayed by the TBS-LFS protocol. On the contrary, both saline- and cocaine-paired groups of mice showed similar hippocampal re-potentiation, which was not detected in naïve mice. These results indicate that the CPP training process has introduced a drug-independent synaptic modification in the hippocampal circuits, which disables LFS to induce depotentiation of TBS-elicited LTP.

Since the drug of choice is not the cause of the CPP task-dependent synaptic modification, shown as hippocampal re-potentiation, we focused on the context where CPP training takes place. During CPP training, the contextual experience in mice can be broken down into differential contextual exposures and *i.p.* injections. We found that mice experiencing *i.p.* injection-free exposures in the CPP apparatus did not develop hippocampal re-potentiation. Moreover, mice receiving *i.p.* injections in the home cage or in a novel cage also did not develop hippocampal re-potentiation. These results suggest that an association between *i.p.* injections and differential contextual exposures at the CPP training stage is required for the development of hippocampal re-potentiation.

Considering using an analogy of Pavlovian conditioning, the differential contextual exposures during CPP training may serve as a conditioned stimulus (CS), which contains a variety of sensory stimuli. The *i.p.* injection can serve as an unconditioned stimulus (US), which brings the distress associated with the injection process [22]. The present study suggests that, after repeatedly pairing, the association of CS-US during the CPP training period emerges, in a cocaine-independent manner, and impacts the synaptic properties of hippocampal circuits. It is revealed as the resistance to LFS-induced depotentiation of TBS-elicited LTP, which keeps hippocampal synaptic transmission remained potentiated. Similarly, enhanced excitatory transmission was also demonstrated in vivo in the hippocampus of mice in a classical Pavlovian conditioning session [23].

The finding that TCS1102, a dual OXR antagonist, can prevent the CPP training-induced hippocampal re-potentiation, suggests that orexins are released during the CPP training process. Orexins can be released through activating hypothalamic orexin neurons under various conditions, such as stress [14, 24, 25], arousal [26, 27] or emotional changes [28, 29]. During the CPP training period, the association of the arousal from differential context exposures and the stress from the paired *i.p.* injection process may activate hypothalamic orexin neurons to release orexins, ultimately modulating hippocampal synaptic properties.

Although the results in a previous CPP test [30] showed that the number of activated orexin neurons was not significantly increased in saline-paired rats, our previous study has demonstrated that orexin at the concentration as low as 1 pM can completely prevent LFS-induced hippocampal depotentiation in mice [12]. It is likely that only a few orexin neurons activated during the CPP training are enough to release the amount of orexins required for modulating hippocampal depotentiation. Since we did not detect hippocampal re-potentiation in the groups of mice placed in home cage or a novel larger cage paired with saline injections, these results suggest little or no orexin release in these conditions. In this regard, the difference in the degree of stress and arousal between the CPP training group and other groups may determine the amount of orexins released. The complexity of the spatial contexts may be an important determinant of the contextual experience. In our experimental preparation, the novel cage is similar to the home cage of mice in terms of material, bedding and shape, except the size. However, the CPP arena has a more restricted spatial compartment with a different wall color and floor structure from the home cage. It provides not only more novel spatial information but also tactile stimulation. Therefore, mice in the CPP apparatus may encounter a higher degree of arousal than in the home cage and, accompanied with the injection stress, have more orexin released, ultimately developing hippocampal re-potentiation.

Similar to our finding here in C57BL/6JNarl mice, LFS also failed to induce depotentiation at hippocampal Schaffer Collateral-CA1 synapses in F344 rats after chronically treated with saline by either *i.p*. injection for 7 days [31] or self-administration for 20 days [32]. In contrast, LFS did induce hippocampal depotentiation in LEW rats, another inbred rat strain, in a concurrent test. F344 rats, compared to LEW rats, are less vulnerable to abusing substances while have a higher responsiveness to stress [33]. It will be interesting to examine whether F344 rats are more vulnerable to the stress-induced arousal than LEW rats and hence have elevated orexins that can prevent LFS-induced depotentiation as we observed in the present study. In this regards, it will be also interestingly to examine whether there is an association between the orexin system activity and the addiction vulnerability among subjects.

## Conclusion

In this study, we have revealed that differential contextual exposures conditioned with *i.p.* injections during the CPP training process can modulate hippocampal synaptic plasticity, revealed as the resistance to depotentiation by TBS-LFS application at hippocampal Schaffer collateral-CA1 synapses. This synaptic modulation occurs regardless of whether CPP is expressed. To the best of our knowledge, this is the first report revealing that the CPP training process per se can modulate hippocampal synaptic plasticity. Importantly, this CPP training-specific hippocampal re-potentiation is mediated by endogenous orexins through both OX_1_Rs and OX_2_Rs.

## Additional file

Additional file 1: Supplementary data. (DOC 848 kb)

## Abbreviations

aCSF: Artificial cerebral spinal fluid
CPP: Conditioned place preference
fEPSPs: Field excitatory postsynaptic potentials
*i.p.*: Intraperitoneal
LFS: Low frequency stimulation
LH: Lateral hypothalamus
LTP: Long-term potentiation
OX_1_Rs: Orexin receptors 1
OX_2_Rs: Orexin receptors 2
OXR: Orexin receptor
TBS: Theta bust stimulation
