# Supplementary material for: Conditioned place preference training prevents hippocampal depotentiation in an orexin-dependent manner
Source: J Biomed Sci. 2017 Sep 6;24:69. doi: 10.1186/s12929-017-0378-0 (PMC5585888; doi:10.1186/s12929-017-0378-0)
Supplement: Additional file 1: — Supplementary data. (DOC 848 kb) [file 12929_2017_378_MOESM1_ESM.doc]

**Supplementary Information**

- **Supplementary Figures: Figure S1-Figure S5.**
- **Supplementary Methods: Immunofluorescence staining**


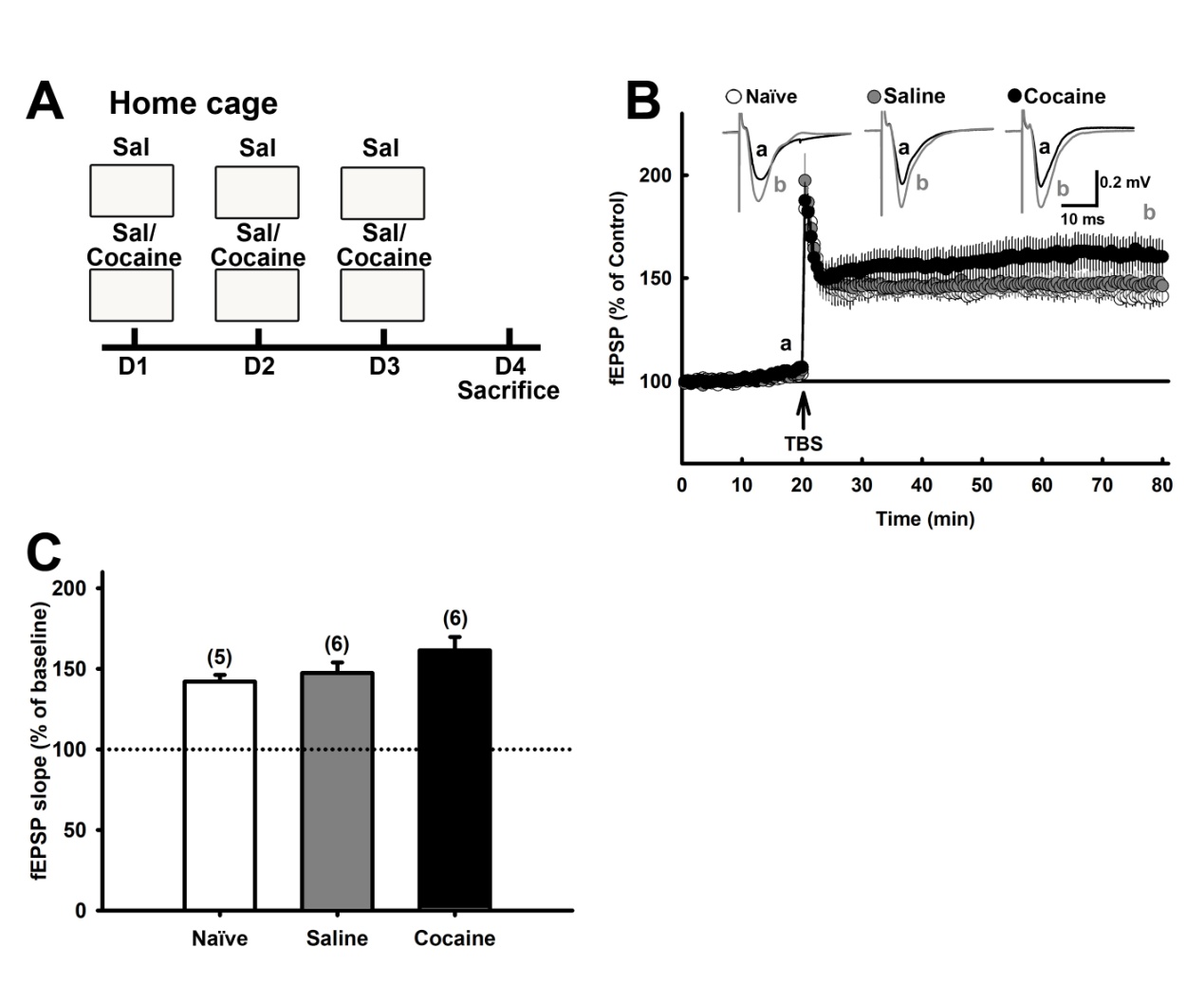


**Figure S1: Home cage pairings with saline or cocaine injections in mice did not affect TBS-induced hippocampal LTP *in vitro*. A:** The schema for a 3-day home cage parings with saline and cocaine (20 mg/kg, *i.p.*) injections under the same protocol as used in the CPP training stage. Hippocampal slices were prepared on Day 4. **B:** Time courses of the slope of fEPSPs recorded before and after TBS in hippocampal slices prepared from untreated naïve mice and mice receiving saline or cocaine injections at their home cage. Inset: Averaged traces of 20 fEPSPs recorded before (a) and 60 min after (b) TBS in each group. **C:** Bar graphs summarize the magnitudes of potentiation of fEPSP slope in the three groups of mice in **B**.


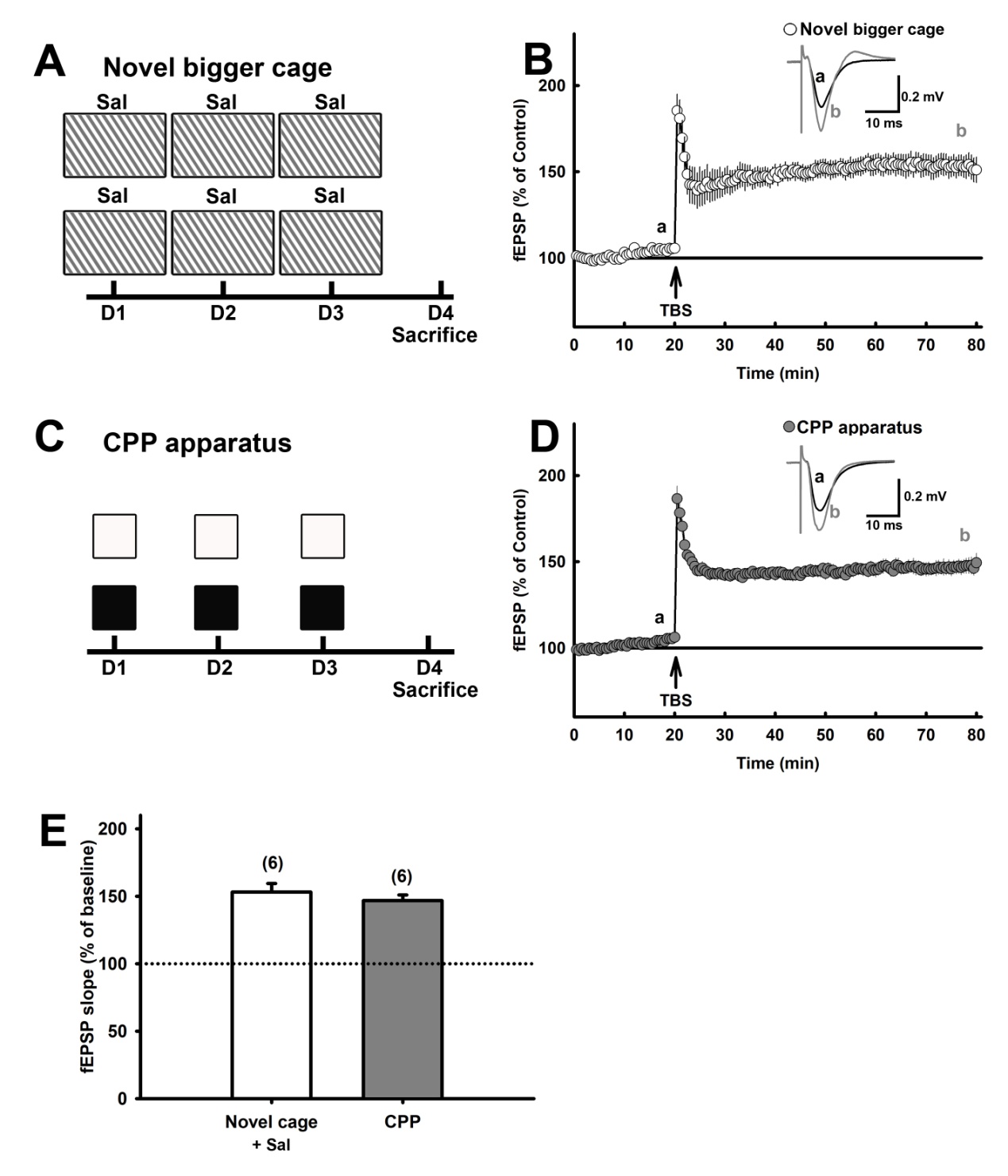


**Figure S2: Novel cage pairing with saline injections or injection-free CPP training did not affect TBS-induced hippocampal LTP *in vitro*. A:** The schema for a group of mice given saline injections twice a day in a novel big cage for 3 days. Hippocampal slices were prepared on Day 4. **B:** Time courses of the slope of fEPSPs recorded before and after TBS in hippocampal slices prepared from mice underwent procedures in **A**. Inset: Averaged traces of 20 fEPSPs recorded before (a) and 60 min after (b) TBS. **C:** The schema for mice underwent *i.p.* injection-free CPP training. Hippocampal slices were prepared on Day 4. **D:** Time courses of the slope of fEPSPs recorded before and after TBS in hippocampal slices prepared from the mice in **C**. Inset: Averaged traces of 20 fEPSPs recorded before (a) and 60 min after (b) TBS. **E:** Bar graphs summarizes the magnitudes of potentiation of fEPSP slope in the two groups of mice in **A** (Novel cage+saline) and **C** (*i.p.*-free CPP).


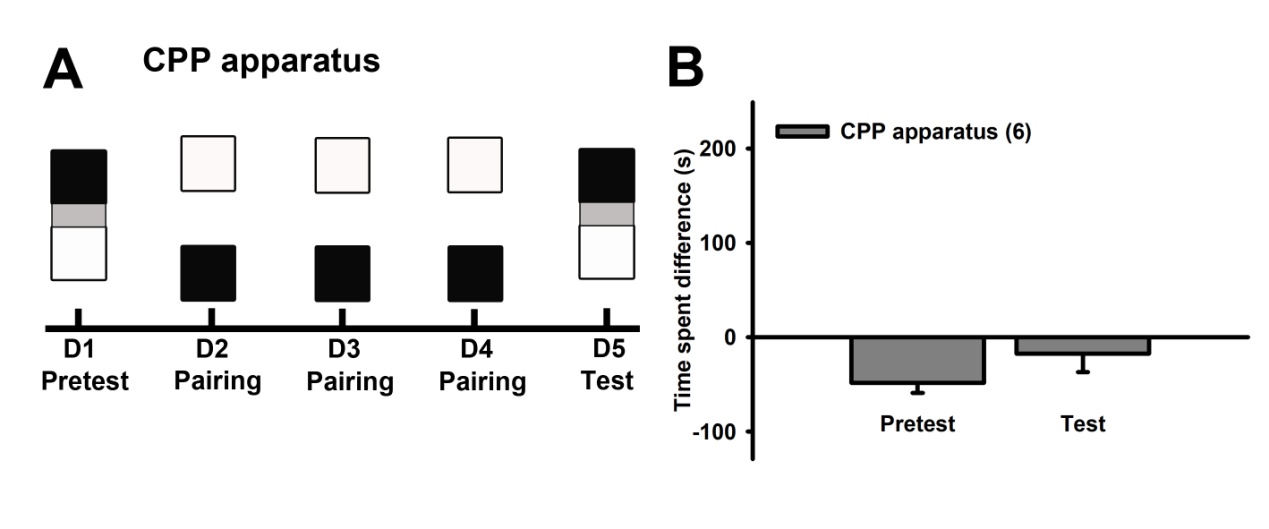


**Figure S3:** **Differential exposures in the CPP apparatus in injection-free mice did not induce preference.** **A:** The schema for a 5-day bias exposures in CPP apparatus with a 3-day training period. Mouse freely moved in CPP apparatus in day 1 (Pretest) and 5 (Test), and paired daily for three days in its non-preferred and preferred arenas (Pairing). **B:** Bar graphs summarize the CPP scores for mice underwent *i.p.* injection-free CPP training.

**
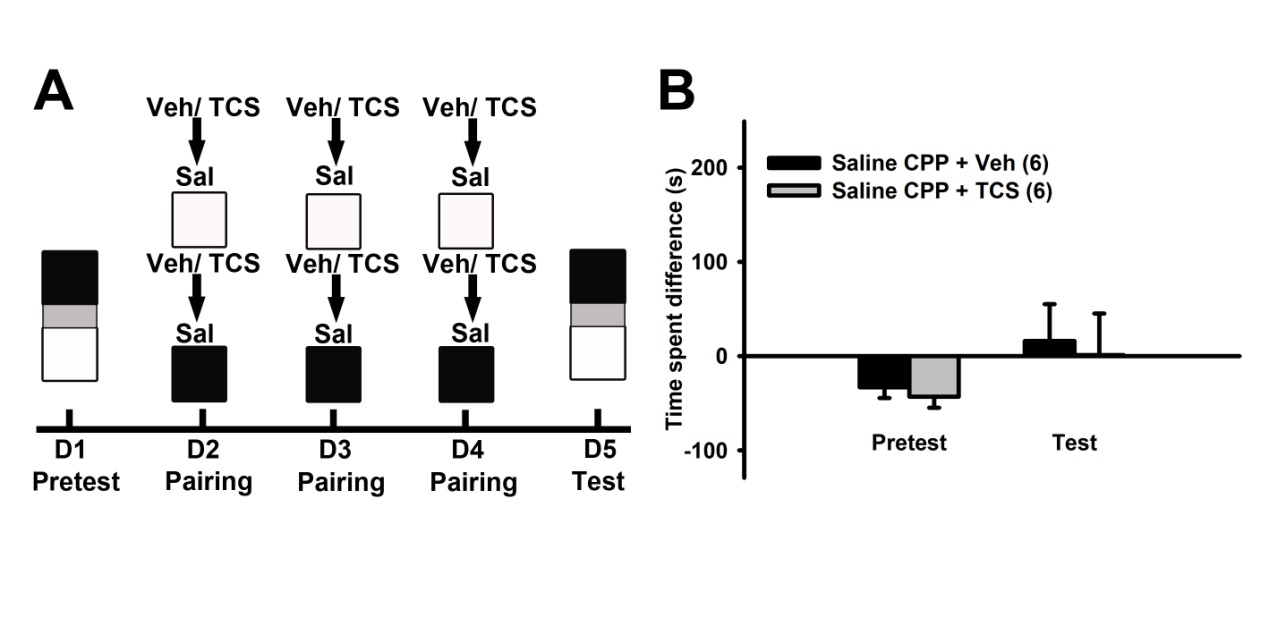
Figure S4: Pretreatment with TCS1102, a dual orexin receptor antagonist did not affect CPP score in mice underwent saline-paired CPP training. A:** The schema for mice given vehicle (Veh) or TCS1102 (TCS, 20 mg/kg, *i.p.*) 30 min prior daily saline-paired CPP training procedures. Mouse freely moved in CPP apparatus in day 1 (Pretest) and 5 (Test), and paired daily for three days in its non-preferred and preferred arenas (Pairing). **B:** Bar graphs summarize the CPP scores for mice given vehicle or TCS groups.


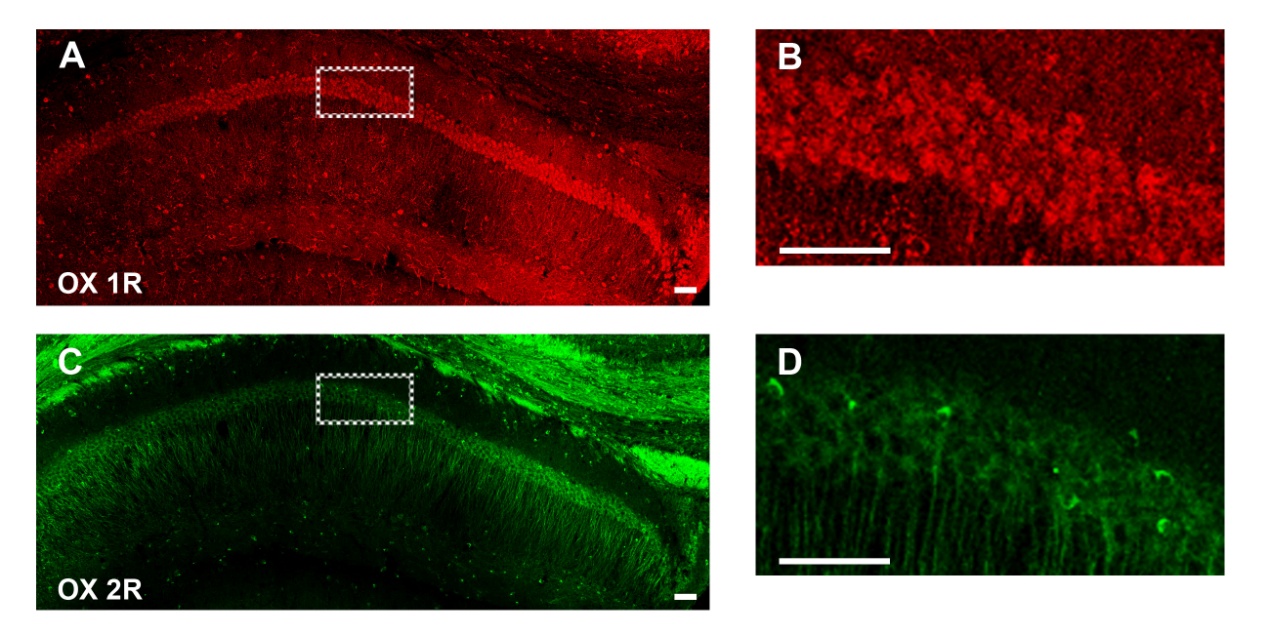


**Figure S5: Immunofluorescence staining for OX_1_Rs and OX_2_Rs in mouse hippocampal slices. OX_1_Rs (red) and OX_2_Rs (green) are present in hippocampal pyramidal neurons including the CA1 region (squares in A and C; B and D) where electrophysiological recordings were performed. A and B:** **OX_1_R** staining. **C and D:** **OX_2_R** staining. Scale bar: 50 μm.

**Supplementary Methods**

**Immunofluorescence staining**

Mouse (n = 3) were deeply anesthetized with isoflurane and perfused transcardially with 15 mL normal saline, followed by 15 mL 4% paraformaldehyde in 0.1 mol/L phosphate-buffered saline (PBS). Subsequently, the brain was removed, trimmed, postfixed in the same fixative for 12 h at 4℃, and then cryoprotected with 30% sucrose for 48~72 h and exchanged sucrose for one time. Frozen coronal sections (30 μm thick) containing the hippocampus were obtained using a freezing microtome (CM 3050S, Leica) and stored in PBS at 4℃. The slices were antigen retrieval by EDTA PBS buffer (1 mM EDTA, pH8.0). Pre-heated the buffer to 80 ℃ and kept temperature to heat the slices for 30 min in EDTA buffer. The slices were washed for 5 min three times with PBS and incubated in freshly prepared sodium borohydride (NaBH4; 1% w/v) in PBS for 30 min. The slices were washed for 5 min three times with PBS and 10 min with PBS containing 0.4% Triton X-100 (PBST). Then the slices were blocked by 10% donkey serum in 2% bovine serum albumin PBST for incubation 60 min. The primary antibodies against the OX1 receptor (a rabbit anti-OX1 receptor polyclonal antibody, 1:200; ab68781 Abcam, Cambridge, UK) were used for incubating overnight at 4℃. After a complete wash with PBS (5 min three times) and PBST (10 min one time), the slices were incubated in the Alexa 594-conjugated donkey anti-rabbit (1:1000; A21207, Life technology) for 2 h at room temperature in the dark. Then washing with PBS (5 min three times) and PBST (10 min one time) again, the primary antibodies against the OX2 receptor (a goat anti-OX1 receptor polyclonal antibody, 1:200; ab189393 Abcam) were used for incubating overnight at 4℃. Washing with PBS (5 min three times) and PBST (10 min one time) again, the slices were incubated in the Alexa 488-conjugated donkey anti-goat (1:1000; A11055, Life technology) for 2 h at room temperature in the dark. Final wash with PBS for 5 min three times and mounted the slices on gelatin coated slides with mounting medium (ProLong Gold Antifade Mountant, P36930, Thermo Fisher Scientific Inc.). All micrographs were captured with an inverted laser scanning confocal microscope (SP5, Leica).
